# The Current State of Knowledge about Essential Oil Fumigation for Quality of Crops during Postharvest

**DOI:** 10.3390/ijms222413351

**Published:** 2021-12-12

**Authors:** Małgorzata Namiota, Radosław Bonikowski

**Affiliations:** Institute of Natural Products and Cosmetics, Faculty of Biotechnology and Food Sciences, Lodz University of Technology, Stefanowskiego 2/22, 90-537 Lodz, Poland; radoslaw.bonikowski@p.lodz.pl

**Keywords:** decay, shelf-life, postharvest storage, antioxidant, volatiles

## Abstract

Prolonging crops’ shelf-life while retaining their high quality is a major issue related to postharvest management. During storage, fruits and vegetables are exposed to microbial attacks, which may cause spoilage. Crop deterioration causes the loss of physical properties and drops in quality and nutritional value. Hence, new techniques to improve the resistance of food products are being explored. One promising technique is fumigation. Essential oils and their constituents, due to their antimicrobial properties, are likely to be used as fumigants, as they are highly volatile, effective in low concentrations, biodegradable, and safe. Papers indicate that some of them can improve their quality by increasing the content of antioxidants. This comprehensive review aims to present the current state of knowledge about the influence of essential oil fumigation on crop quality. It covers antioxidant capacity, the content of some bioactive compounds, physicochemical properties, decay properties, and sensory attributes of fruits and vegetables treated with essential oil vapors. The review indicates that this technique might be an interesting field for further exploration due to the promising results presented in the studies. Moreover, the review presents major objectives for current studies and indicates a lack of recent papers in this field.

## 1. Introduction

Postharvest storage faces problems with postponing losses of fruits and vegetables due to decay. Decay is mainly triggered by fungal plant pathogens that cause several types of rot, mold, and anthracnose disease development and the production of mycotoxins [1]. The resistance of fruits and vegetables to pathogens is dependent on, among others, the properties of crops (e.g., the thickness of the skin), quality of the freshly picked crops, cultural practices, field nutrition, type of harvesting, storage conditions, and postharvest treatment [2]. To control postharvest diseases, it is common to apply synthetic fungicides; however, biocontrol agents are no less popular [1].

**Figure 1:**
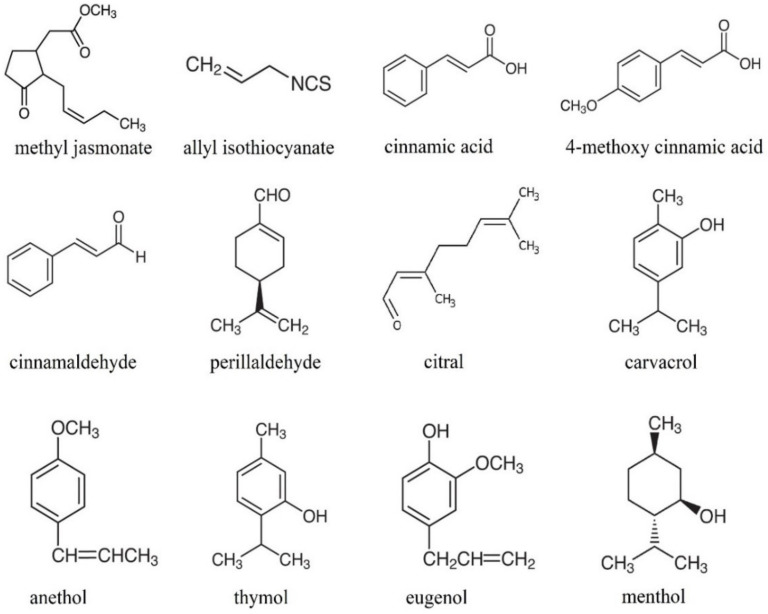
Chemical formulae of some essential oils’ constituents.

The growing demand for eco-friendly solutions in the food industry forces technologists to design methods using biodegradable, natural products for food preservation. Hence, the industry is turning its eyes to essential oils. Although essential oils (EOs) have been known since the dawn time as a rich source of antioxidants and antifungal and antibacterial substances, their application in food preservation is not very common. Fumigation with essential oils may be applied to change crops’ biochemical, microbial, and physicochemical parameters. Because of their high activity in the vapor phase, essential oils are likely to be used as fumigants. Due to the need to introduce safe and effective preservatives in low concentrations, the application of essential oils seems to be justified. It was demonstrated that essential oils applied directly to crops effectively inhibited fungal growth, fungal spore germination, anthracnose incidence, and rotting [3,4]. The mechanism of action of essential oils is believed to be connected with the membrane disruption by EO compounds due to their low molecular weight and lipophilicity [5]. Volatile compounds of essential oils diffuse across cell membranes and, as a result, induce biological reactions in tissues [6]. Some of the essential oils’ compounds are presented in Figure 1. It should be noticed that EO scents highly influence the sensory attributes of fruits and vegetables. Therefore, they should be matched adequately to the type of the crop in order to complement its sensory nature.

In the studies, different techniques of treatments with the use of essential oil vapors were presented. Ding et al. [7] used airtight containers, and volatiles were placed on a filter paper and the processing conditions were 16 h and 23 °C. Harpaz et al. [8] determined the effect of thyme oil vapors on sea-bass fish. The researchers used a Styrofoam box with ice on the bottom and above the sack, and essential oil was dropped on the side of the bag. Fish were stored at 0 to 2 °C. Polystyrene containers with snap-on lids were used in the fumigation of *Rubus idaeus* L. (raspberries), but the volatiles were placed in beakers. The vaporization conditions were 20 °C and 16 h [9,10]. The same conditions were repeated for the *Fragaria x ananassas* Duch. (strawberries) [11], *Rubus* L. (blackberries), and *Vaccinium corymbosum* L. (blueberries) fruits [12,13]; however, in those cases, the temperature was equal to 10 ºC. For the preharvest treatment of *Lactuca sativa* L. var. *longifolia* (romaine lettuce) leaves, methyl jasmonate solutions dissolved in methanol were sprayed and covered with a plastic cover for 1 h [14]. During the subsequent years, in most of the experiments, polystyrene and airtight containers were used. Placing the crops in polypropylene and polyethylene boxes is popular in modern studies, such as Qu et al. [15], Hu et al. [16], T. Jiang et al. [17], or Gao et al. [18]. However, Santoro et al. [19] used diffusers made by mixing the essential oil with sterilized deionized water, Tween 20, and agar on a Petri dish. Many studies also focus on adding essential oils to modified atmosphere packages (abbreviated as MAP) to increase their activity [20].

Essential oils seem to be effective additives to postharvest crops, and various techniques are used. However, as the literature indicates, the type of essential oil, its concentration, and the treatment conditions should be adjusted adequately to the fumigated crop. This paper aims to bring into focus the current state of knowledge about the effects of fruit and vegetable fumigation with essential oils and their constituents. It focuses on biochemical, physicochemical, and microbial parameters and the decay properties of crops treated with plant volatiles. The positive influence of fumigation might be beneficial for the food industry as it could reduce food losses by extending the crops’ shelf-life. Some information about the effects of essential oils and their constituents is gathered in Table 1, below Section 8.

## 2. Effects on Non-Nutrient Compounds and Antioxidant Capacity

This section describes the effects of essential oils fumigation on crops’ biochemical properties: non-nutrient compounds (phenolic, flavonoids and anthocyanins content) and antioxidant capacity in terms of free radicals and enzymatic activity.

### 2.1. Phenolics, Flavonoids, and Anthocyanins

Phenolic and flavonoid compounds significantly preserve food products’ quality due to their antioxidant properties concerning metal chelating and lipid peroxidation inhibition by scavenging superoxide anions and hydroxyl radicals [21,22]. Therefore, the amounts of phenolics, flavonoids, and anthocyanins present in plants are connected with the radical scavenging properties and, consequently, resistance to spoilage and microbial attacks. Several essential oils are composed of phenolic compounds in a considerable concentration. The richest and most common are thyme (*Thymus vulgaris* L.) essential oil containing thymol, clove (*Syzygium aromaticum* L.) essential oil containing eugenol, and oregano (*Origanum vulgare* L.) essential oil containing carvacrol. There are many studies about the essential oils’ capability to enhance antioxidant capacity by keeping high levels of phenolic compounds in the fruits’ composition.

One of the most popular volatile compounds used as an enhancer of the antioxidant capacity of crops is methyl jasmonate because it is produced in plants in stressful conditions and plays a role in defense mechanisms [23]. Hence, it was assessed for its effects on secondary metabolites. *Prunus persica* L. fruits (peaches) treated with methyl jasmonate vapors were characterized with higher (than control) total phenolics content after 2, 4, and 6 days of storage [24]. Additionally, combining methyl jasmonate with ethanol gave even more interesting results for *Myrica rubra* Seib and Zucc. (Chinese bayberries) inoculated with *P. citrinum* [25]. This study showed that both substances enhance total phenolics, anthocyanins, and flavonoids content; however, the best results were obtained when combining those two. The conclusion made by the researchers was that methyl jasmonate and ethanol might possess additive effects.

What is more, studies prove that a similar effect can be obtained in plants and mushrooms. According to Meng et al. [26], *Agaricus bisporus* L. (button mushroom) fruiting bodies fumigated with methyl jasmonate were characterized with total phenolics content higher than that in non-fumigated crops after 2, 3, 4, 5, and 6 days of storage. After 7 days, those values were about equal. They also had a higher value of flavonoids after 2, 3, 4, and 5 days of storage. Another study using methyl jasmonate and, as far as we are aware, the first studies assessing total phenolics content in crops processed with the addition of natural volatile compounds, was Chanjirakul et al. [9]. This one focused on *Rubus idaeus* L. fruits treated with methyl jasmonate, allyl isothiocyanate, tea tree (*Melaleuca alternifoli* L.) essential oil, and ethanol. Of all those substances, methyl jasmonate gave the highest values of total phenolics and anthocyanins; however, samples treated with tea tree essential oil and ethanol were characterized with values higher than the control sample. Neither phenolics nor anthocyanins content increased due to allyl isothiocyanate treatment. Despite those results, another paper on considered compounds was prepared, focusing on the fumigation of *Vaccinium corymbosum* L. fruits [27]. Again, the results showed that allyl isothiocyanate-treated samples contained fewer phenolic compounds and anthocyanins than non-treated ones.

From the terpenoids used as antioxidant capacity enhancers, the most common are menthol, thymol, eugenol, and carvacrol. The first three were tested by C. Y. Wang et al. [11] with regard to phenolic and anthocyanin content in *Fragaria x ananassas* Duch. fruits. The authors presented results showing an increase in both total phenolics and anthocyanins content during 3 weeks of storage. The highest values were obtained when *Fragaria x ananassas* Duch. fruits were treated with thymol, then menthol and eugenol, respectively. The values for phenolics were maintained at the same level in the control samples and were slightly higher for anthocyanins. Results for specific phenolic compounds and anthocyanins showed increased quercetin 3-glucoside and quercetin 3-glucuronide, kaempferol 3-glucoside, cyanidin 3-glucoside, cyanidin 3-glucoside succinate, and pelargonidin 3-glucoside succinate content for all the essential oils’ compounds with respect to the control samples. The amounts of *p*-coumaric acid, ellagic acid, ellagic acid glucoside, and pelargonidin 3-glucoside increased due to menthol and thymol treatment. However, they decreased in eugenol-treated samples, and the amount of kaempferol 3-glucuronide increased due to menthol and thymol treatments and kept the same value for eugenol treatment—compared to the non-treated samples. Effects obtained when using menthol in a fumigation process were consistent with Qu et al. [15] and Sellamuthu, Sivakumar, et al. [28]; however, in those two papers, peppermint (*Mentha piperita* L.) essential oil was also used. In the study by Qu et al. [15], which assessed the effects on *Agaricus bisporus* fruiting bodies, the content of phenolics in the control sample dropped during the following days of storage, while for those treated with peppermint essential oil, it was rising. It should be mentioned, though, that the fruiting bodies of mushrooms were stored together in a container—60 mushrooms in one box. The researchers chose mushrooms of uniform size but as they grow quickly, it is not possible to always obtain a harvest of uniform crops. Furthermore, because the samples were taken out from the box every 2 days, every time one was removed, the actual concentration of essential oil per one sample changed. Therefore, although the researchers stated that all samples in one lot were fumigated by the same concentration of essential oil, the authors of this review must question the validity of this statement. What is more, because every time a sample was taken from the container, some of the essential oil evaporated from it.

Sellamuthu, Sivakumar, et al. [28] assessed the effects of fumigating *Persea americana* Mill. (avocado) fruits with thyme, peppermint, and citronella (*Cymbopogon nardus* L.) essential oils. The highest values for total phenolics content were obtained with thyme essential oil, followed by peppermint essential oil, and then the citronella essential oil from those substances. Since menthol is the main constituent of peppermint essential oil, this compound could be mainly responsible for the effects.

Predominately, studies on the use of thyme essential oils and thymol have been carried out. Valero et al. [29] prepared an experiment that analyzed the properties of *Vitis vinifera* L. (table grape) fruits stored in a modified-atmosphere package combined with thymol and eugenol. Fruits stored in the atmosphere with reduced oxygen together with the vapors of essential oil compounds were divided into pulp and skin and analyzed for total phenolics content. The results for the fruits’ skin after 14 days of storage showed an increase in the level of phenolic compounds for the samples treated with 75 and 150 µL of eugenol and 150 µL of thymol, whereas for the one treated with 75 µL of thymol and for the control sample, a decrease was noticeable. In the following days, all of the samples suffered a decrease in total phenolics. It was shown that the smallest drop in total phenolics content was obtained with the addition of 150 µL of eugenol, followed in order by the treatments with 150 µL of thymol, 75 µL of eugenol, 75 µL of thymol, and then control sample, respectively. This sequence is consistent with results for the fruits’ pulp; however, in this test, all of the treated samples experienced increases in total phenolics content during the entire period of storage, obtaining a maximum after 56 days.

Similarly, thymol vapors were responsible for a high level of total anthocyanins content in *Fragaria x ananassas* Duch. fruits compared to non-treated control samples [30]. Since eugenol and thymol are ubiquitous constituents in popular, herbal essential oils, there has been more research on determining their effectiveness in this considered matter. Thyme essential oil alone in MAP was also analyzed in research provided by Sellamuthu, Mafune, et al. [31]. These papers prove that combining thyme essential oils with a modified atmosphere enhances antioxidant compounds better than MAP alone. Two cultivars of *Persea americana* Mill. fruits stored in MAP for 18 days were characterized with high phenolics and flavonoids content.

There are also reports on the positive influence of fumigation with thyme essential oils on phenolic compound maintenance in mushroom fruiting bodies. Gao et al. [18] studied the chemical composition of postharvest *Agaricus bisporus* fruiting bodies after fumigation with 10 µL of clove, thyme, and cinnamon (*Cinnamomum zeylanicum* L.) essential oils. The experiment showed that after 16 days of storage, a decrease of 200 mg/kg in a control sample occurred, while for the clove essential oils, the decrease equaled 100 mg/kg. For thyme and cinnamaldehyde, it equaled the same value. However, during the first 4 h of storage, increases in total phenolic compound content were observed in all of the treated samples. In T. Jiang et al. [17], the antioxidant activity of *Lentinus edodes* L. (shiitake mushrooms) fruiting bodies fumigated with cinnamaldehyde and clove and thyme essential oils in the same concentration was assessed. The results for total phenolics content showed that while a control sample suffered a significant decrease, a lower decrease was observed in the rest of the samples. Again, the lowest rate of decrement in phenolics content occurred in samples treated with volatiles. Perumal et al. [32] presented the effects of fumigating two varieties of *Mangifera indica* L. (mango) fruits with thyme, clove, and cinnamon essential oils. The fruits were infected with spore suspensions of *C. gloeosporioides* and *L. theobromae*. The experimental results showed higher values of total phenolics content in all of the treated samples than in the non-treated control sample. The highest value was obtained using thyme essential oil. After 4 days of storage, it was equal to 2.16 ± 0.05 mg/g (cv. “Banganapalli”) and 2.72 ± 0.02 mg/g (cv. “Totapuri”), while for the control sample, it was equal to 1.13 ± 0.05 mg/g (cv. “Banganapalli”) and 1.72 ± 0.04 mg/g (cv. “Totapuri”). Results for samples treated with clove and cinnamon essential oils were lower but still higher than those for the non-treated samples.

Moving to other examples, Jin et al. [33] assessed the effects of essential oil treatment on antioxidant capacities of *Myrica rubra* Seib and Zucc. fruits. Samples were treated with the main constituents of known essential oils: carvacrol, cinnamaldehyde, perillaldehyde, and linalool vapors. After 3 days from the fumigation process, there was a significant increase in total phenolic content in all of the treated samples—from 290.89 ± 8.13 mg/100 g of fresh weight to a minimum 302.15 ± 3.33 mg/100 g of fresh weight (in linalool-treated sample) and maximum 358.64 ± 4.01 mg/100 g of fresh weight (in carvacrol-treated sample), while in control samples there was a visible decrease, to 271.15 ± 9.06 mg/100 g of fresh weight. During the following days of storage, a decrease in total phenolics content was observed in all of the samples.

The increase in total phenolics and anthocyanins also occurred in *Vaccinium corymbosum* L. (cv. “Duke”) fruits treated with carvacrol, anethole, cinnamaldehyde, and perillaldehyde vapors [13]. Such results suggest that these volatiles are capable of promoting phenols and anthocyanins. This study denied a positive effect of fumigation with linalool and *p*-cymene on the level of total flavonoids; for samples treated with either of those two compounds, it was lower than for the control sample. Additionally, there was a slight decrease tin of total anthocyanins content in *Vaccinium corymbosum* L. fruits treated with cinnamic acid compared to the non-treated sample. Those results were directly correlated to the specific anthocyanins and phenolics content in *Vaccinium corymbosum* L. fruits. All of the tested compounds enhanced chlorogenic acid, delphinidin 3-galactoside, cyanidin 3-galactoside (anethole and linalool gave twice that of the control), delphinidin 3-arabinoside (anethole gave almost twice the value of control), petunidin 3-arabinoside, malvidin 3-galactoside, and malvidin 3-arabinoside content in *Vaccinium corymbosum* L. fruits. The amount of myricetin 3-arabinoside decreased for cinnamic acid, cinnamaldehyde linalool, and *p*-cymene, and the amount of kaempferol derivative of petunidin 3-glucoside decreased for cinnamic acid. Neither garlic (*Allium sativum* L.) essential oil nor rosemary (*Rosmarinus officinalis* L.) essential oil enhanced *Fragaria x ananassas* Duch. fruits’ total phenolics and flavonoid content [34]. On the contrary, treated samples possessed lower levels of those compounds than the non-treated control samples. Additionally, in the control samples, the concentration of phenolic compounds increased during storage, while for the treated samples, only decreases were observed. It should be noted, however, that essential oils used in this research were applied topically in the liquid state. With such a technique, the essential oils must be used in high concentrations—700 and 1700 µL/L in this study. Therefore, the costs of this method are huge. Moreover, the efficiency is rather low due to the quick vaporization of the essential oils.

Some of the aforementioned studies hypothesize that several essential oil compounds may trigger the signaling pathways to activate defense mechanisms that stimulate tissues to increase primary and secondary antioxidants, such as phenolic compounds [31]. Another possible explanation is connected with the observation that in those fruits where the increase in phenolics occurs, the increase in phenylalanine ammonia-lyase (PAL) activity is also observable. It happens because PAL is an enzyme involved in the biosynthesis of the phenolic compounds [35], and assuming that introducing essential oils to the plant’s tissues may enhance the antioxidant’s capacity due to the increased amount of this enzyme, the increases in phenolic compound content should be observed [33]. Furthermore, phenolics content is connected with the phenylalanine ammonia-lyase enzyme, also according to the fact that this enzyme catalyzes reactions involved in the biosynthesis of phenolic compounds [36]. Hence, with high values of phenolics observed in the studies mentioned above, high PAL activity values should be observed. This hypothesis will be analyzed in Section 2.2.

### 2.2. Enzymatic Activity

The abbreviations used in this section are as follows: superoxide dismutase (SOD), catalase (CAT), peroxidase (POD), ascorbate reductase (APX), glutathione peroxidase (GSH-POD), ascorbate peroxidase (AsA-POD), guaiacol peroxidase (G-POD), monodehydroascorbate reductase (MDHAR), dehydroascorbate reductase (DHAR), glutathione reductase (GR), and polyphenol oxidase (PPO).

Enzymatic activity is highly responsible for cellular protection due to oxidation. In particular, SOD is a primary warrior in the battle with reactive oxygen species; it catalyzes dismutases of two superoxide anion radicals to hydrogen peroxide and O_2_ [37], and due to this fact, it is called a perfect antioxidant. Peroxidase and catalase are activated due to physiological stress from, for example, microbial or pest attacks [38].

However, the high activity of those enzymes is responsible for unfavorable changes in the plants’ texture, taste, or scent and may cause browning [39]. Together with superoxide dismutase, they help to remove reactive oxygen species [40].

Many studies suggest that some essential oils are capable of enhancing the enzymatic activity of peroxidase and catalase. For example, thyme essential oil vapor induced activity of POD and CAT in *Persea americana* Mill. [28] and *Mangifera indica* L. fruits [32]. Additionally, enhancement of the peroxidase activity in fumigated crops was achieved using citronella [28], clove, and cinnamon essential oils [32]. On the other hand, Jin et al. [24] reported an increase in polyphenol oxidase and peroxidase activity in *Prunus persica* L. fruits due to methyl jasmonate vapor, but at the same time, the activity of catalase decreased. Studies on specific peroxidases in *Rubus idaeus* L. fruits fumigated with methyl jasmonate, tea tree essential oil, and allyl isothiocyanate showed high increases in GSH-POD, AsA-POD, and G-POD activity due to methyl jasmonate treatment, a slight increase due to tea tree essential oil treatment, and a serious decrease due to allyl isothiocyanate treatment [9]. That study indicated that of the enzymes engaged in a Foyer-Halliwell-Asada pathway, the activities of MDHAR, DHAR, and GR were highest for methyl jasmonate-treated samples, followed by tea tree essential oil-treated samples, allyl isothiocyanate-treated samples, and control samples, respectively. The activity of all of those enzymes dropped during storage; however, it remained at higher levels in methyl jasmonate and tea tree essential oil-treated *Rubus idaeus* L. fruits than in non-treated ones. That was connected with the levels of glutathione, ascorbate, and dehydroascorbate in the fruits’ extract.

A study on *Myrica rubra* Seib and Zucc. treated with carvacrol, cinnamaldehyde, perillaldehyde, and linalool showed that all volatiles enhanced total peroxidase, catalase, and ascorbate peroxidase activities for the duration of storage [33]. However, carvacrol and cinnamaldehyde increased APX activity from 28.23 µmol of ascorbate/min to 43.66 and 36.19 µmol of ascorbate/min, respectively, after 3 days of storage, while the control sample possessed a level of APX equal to 18.50 µmol of ascorbate/min. Therefore, while this activity dropped in non-treated samples, it increased in samples treated with carvacrol and cinnamaldehyde. On the other hand, there were no significant increases in enzymatic activity in *Fragaria x ananassas* Duch. fruits treated with goldenrod (*Solidago canadensis* L.) essential oil vapors, except for β-1,3-glucanase activity [41].

Such increases in POD and PPO activities have not yet been observed for *Agaricus bisporus* L. fruiting bodies. Their levels were determined after processing fruiting bodies with peppermint essential oil vapors [15], cinnamaldehyde, clove, and thyme essential oil vapors [18], 4-methoxy cinnamic acid vapor [16], methyl jasmonate vapors [26], and chitosan nanoparticles containing bitter orange (*Citrus aurantium* L.) essential oil [42]. According to those results, the concentrations of proteins catalyzed by peroxidases or polyphenol oxidase were smaller in processed samples than in control samples, or the results were not statistically significant. Therefore it was seen that essential oils either did not influence enzymatic activity or inhibited it significantly.

On the other hand, the determination of ascorbate peroxidase and glutathione reductase levels in another variety of mushroom fruiting bodies—*Lentinus edodes* L. fumigated with cinnamaldehyde, clove, and thyme essential oils—suggested that this enzyme activity could be enhanced due to the presence of plant volatiles [17]. APX activity increased enormously in samples until 10 days after storage, and the levels of activity were about three times higher in the cinnamaldehyde-treated sample than in samples before treatment, followed by the thyme essential oil treatment, which was about 2.5 times higher. In turn, the GR activity in samples treated with cinnamaldehyde rose until 15 days after storage, reaching values about 2.5 times higher than those before treatment. Fumigation with thyme essential oil resulted in 30% stronger GR activity in this sample than in a non-treated sample [17]. Both of those enzymes play essential roles in the glutathione-ascorbate cycle, so their activity is crucial for detoxifying hydrogen peroxide. This study showed that catalase activity was induced after processing and reached a maximal level after 5 days of storage, and then it gradually decreased.

When it comes to superoxide dismutases, their activity was visibly higher in all the treated crops with respect to the control samples in most of the considered studies. For example, fumigation of *Rubus idaeus* L. fruits, *Prunus persica* L. fruits, and *Agaricus bisporus* L. fruiting bodies with methyl jasmonate enhanced superoxide dismutase activity throughout the period of storage [9,24,26]. Similarly, treatments with peppermint oil strongly increased SOD levels in *Agaricus bisporus* L. sporophores [15]. Together with citronella and thyme essential oils, the fair effects of this oil were observed in avocado [28]. Moreover, treatments with thyme, clove, and cinnamon essential oil induced SOD activity in mango fruits [32]. A similar set of volatiles (however, with cinnamaldehyde used instead of cinnamon essential oil) helped maintain superoxide dismutase activity in shiitake mushrooms after 10 or more days of storage [17].

A significant increase was also observed with carvacrol treatment of *Myrica rubra* L. fruits, in which the level of SOD increased after 3 days of storage and, even though it then started to drop, it remained at higher levels than in the control samples during the whole experiment [33]. According to this study, cinnamaldehyde treatment provided moderate effects and perillaldehyde and linalool, minor effects. Studies also showed that fumigation with allyl isothiocyanate decreases SOD activity in *Rubus idaeus* L. fruits [9] but increases SOD activity in *Morus alba* L. (mulberry) fruits, regardless of the concentration [43]. Interestingly, adding bitter orange essential oil to chitosan nanoparticles helped to obtain a higher level of superoxide dismutase activity than in control samples and samples treated with nanoparticles without essential oil [42].

As mentioned in Section 2.1., the enzyme phenylalanine ammonia-lyase plays a vital role in the pathway of phenolic compound synthesis. However, it is also responsible for catalyzing the trans-elimination of ammonia from L-phenylalanine [44] and responds to biotic and abiotic stresses that occur in plants. All the studies in which both PAL activity and total phenolics were determined showed that when enzymatic activity rises, the amount of phenolic compounds rises, which is consistent with the theory. Thus, it can be concluded that the high activity of phenylalanine ammonia-lyase promoted the increase in total phenolics content due to vapors of thyme essential oil [18,28,31], peppermint essential oil [15,28], methyl jasmonate [24,45], clove essential oil [18,32], and cinnamaldehyde [18].

### 2.3. Antioxidant Activity against Free Radicals

Free radicals, mostly oxygen reactive species, cause cell damage; they are incredibly deleterious and responsible for the pathogenesis of diseases. Therefore, free radical scavenging is considered to be one of the best indicators of antioxidant activity. In plants, it is related to the content of phenolic and flavonoids compounds and some nutrient compounds such as ascorbic acid, tocopherols, and carotenoids [46].

Studies suggested that treating *Vaccinium corymbosum* L. fruits with allyl isothiocyanate vapor lowers the radical scavenging capacity against DPPH radicals [47]. Additionally, according to the electron spin resonance evaluation, it promoted the production of DPPH radicals. On the other hand, antioxidant capacity was enhanced by the cinnamon essential oil, increasing DPPH scavenging activity in *Vitis vinifera* L. fruits from 30% before treatment to 50% after 10 days of storage when applied in a concentration of 0.588 g/L [48]. At the same time, the radical scavenging activity in the control sample was equal to about 25%. However, samples treated with lower concentrations of essential oil possessed weaker activity, of about 40% with 0.392 g/L and 20% with 0.196 g/L. Following 5 days of storage, all of the values met at a similar point of about 20%. Higher DPPH scavenging activity was also shown in *Fragaria x ananassas* Duch. treated with thymol vapors, determined by electron spin resonance [11]. The experiment showed that the strongest activity was obtained using thymol, then menthol and eugenol, respectively. In *Rubus* L. and *Fragaria x ananassas* Duch. fruits treated with methyl jasmonate, allyl isothiocyanate, and tea tree essential oil, DPPH activity was the highest in methyl jasmonate-treated samples according to the fact that it needed the lowest amount of antioxidants to quench 50% of the initial DPPH value (values expressed as ED50) [12]. The next in line were as follows: control sample, tea tree essential oil-treated sample, and allyl isothiocyanate-treated sample for *Rubus* L. fruits. For *Fragaria x ananassas* Duch. fruits, the results were as follows: control sample, allyl isothiocyanate-treated sample, and tea tree oil-treated sample. It was also shown that in the case of *Fragaria x ananassas* Duch. fruits, values of ED50 were higher, probably because they possess lower amounts of secondary antioxidants. Methyl jasmonate-treated samples had the highest percentage value of inhibited DPPH radicals in *Fragaria x ananassas* Duch. fruits after 7 days of storage, followed by the tea tree essential oil, control sample, and allyl isothiocyanate. Seven days of storage did not influence the antioxidant capacity much. In the case of *Rubus* L. fruits, all treated samples had lower radical signals for DPPH than those of the control after 7 days of storage; however, they slightly decreased after the following 7 days. A similar tendency was also observed according to the ABTS* assay. ABTS scavenging activity was higher in blackberries than in strawberries; it decreased in all the cases except the methyl jasmonate-treated samples of *Fragaria x ananassas* Duch. fruits.

Methyl jasmonate also enhanced the DPPH scavenging activity of *Myrica rubra* Seib and Zucc. fruits, increasing its capacity from 50% before treatment to 70% after 3 days of storage [45]. In comparison, this activity in the control sample after 3 days of storage did not change. During the following days, the DPPH scavenging capacity decreased in both samples. That is also consistent with the increased values for total phenolics and flavonoids in the treated samples in this experiment. Kim et al. [14] showed similar effects by determining the correlation between the total phenolics content and the DPPH scavenging capacity in *Lactuca sativa* L. var. *longifolia* on a level of R2 = 0.989. Methyl jasmonate in this study enhanced radical scavenging activity against both ABTS* and DPPH radicals in romaine lettuce.

*Lentinus edodes* L. fruiting bodies fumigated with clove, cinnamaldehyde, and thyme oils suffered a vast drop in scavenging capacity after 5 days of storage [17]. However, during the following days, the capacity rapidly increased in the treated samples, reaching a maximum for the cinnamaldehyde-treated sample on day 15 (15% higher than before the treatment), in the thyme essential oil-treated sample on day 20 (3% higher than before the treatment) and in the clove essential oil-treated sample on day 20 (1% higher than before the treatment). On the other hand, radical scavenging capacity in control samples steadily dropped through the storage period, and on day 20, it was lower than before the treatment, by about 24%. That is consistent with the results for total flavonoids content, where the cinnamaldehyde-treated samples possessed the highest level of those compounds and the control samples, the lowest. According to the ABTS* scavenging activity determination, different results were obtained—the values dropped significantly; however, all of the treated samples of *Lentinus edodes* L. fruiting bodies possessed higher activity than the controls.

## 3. Effects on Micronutrient Compounds

The amount of ascorbic acid is dependent on the intensity of the oxidation process by ascorbate oxidase due to enzymatic activity, and its high value may influence the spoilage and ripening of crops [16,42]. Due to this fact, its concentration in plants is dependent on the storage time, the temperature during storage, and the amount of oxygen in the atmosphere. Hence, methods of reducing oxygen availability, such as coatings or modified atmosphere packages, are believed to be appropriate for maintaining high vitamin C levels [49]. It was proven that injecting essential oils of *Origanum minutiflorum* L., *Juniperus excelca* L., *Melaleuca Leucadendron* L., *Melaleuca armillaris* L., and carvacrol into the rats’ bloodstream resulted in an increasing level of vitamin C in erythrocytes [50,51]. When it comes to plants, it has been suggested that inhibition of vitamin C loss in horticultural crops processed with essential oils is an effect of protecting phenolic compounds present in essential oils’ compositions [52].

The amount of vitamin C in *Solanum lycopersicum* L. (tomato) fruits enriched by oregano essential oil vapor decreased after 1 week of storage from 0.087 mg/g of fresh weight to 0.062 mg/g of fresh weight [53]. After the second week, the level of ascorbic acid in fumigated *Solanum lycopersicum* L. fruits was equal to 0.159 mg/g of fresh weight—twice the initial value—and control samples possessed 0.060 mg/g of fresh weight of ascorbic acid. Therefore, it was observed that in normal conditions, the content of vitamin C decreases due to oxidation processes; however, essential oil vapors are capable of maintaining or even increasing the level of vitamin C, probably in connection with the changes in the level of ascorbate-glutathione pathway enzymes. A study on the influence of thyme and savory (*Satureja montana* L.) essential oil vapors on vitamin C content showed that the treatment slows down the ascorbic acid loss in *Prunus persica* var. nucipersica (nectarines) fruits [19]. They maintained higher levels of this compound than in control samples after 14 and 28 days of storage. It was observed on day 14 that 10% thyme essential oil vapor assured about 23 mg of vitamin C/kg. At the same time, other treatments did not show any significant differences. After the next 14 days of storage, all of the values decreased below 10 mg/kg; however, the control sample possessed the lowest level of vitamin C—close to 0.

Thyme and savory essential oils caused a slighter decrease in vitamin C content in the fumigated samples of *Prunus avium* L., cv Ferrovia (sweet cherry) fruits than in the non-treated samples [54]. In this experiment, fruits were stored in two conditions—28 days in a cold room and then, 3 days in shelf-life temperature. It was similar to keeping the crops in a refrigerated warehouse and moving them into the regular shop. Similar results were observed after fumigating *Prunus persica* L. fruits; after 14 days of storage, the control sample possessed the highest level of vitamin C, while treatments with savory and thyme essential oils caused significant drops in vitamin C content [19]. During the following 14 days, the levels dropped below 5 mg of vitamin C/kg. Therefore it suggests that none of the volatiles is universal and should be matched to the treated plant, based on thorough research. In addition, a slowing down of the decrease in vitamin C was observed in *Agaricus bisporus* L. fruiting bodies treated with 4-methoxy cinnamic acid [16], clove, thyme essential oils, and cinnamaldehyde [18].

What is more, thymol had a positive effect on maintaining high levels of ascorbic acid when used as a fumigant for *Fragaria x ananassas* Duch. fruits [30], in contrast to thyme essential oil, which caused a decrease in vitamin C in *Fragaria x ananassas* Duch. fruits [55]. Treatment with methyl jasmonate was proven to help maintain ascorbic acid content by maintaining higher levels of ascorbate and dehydroascorbate in raspberries than in the control samples; however, tea tree oil-treated samples also possessed higher values [9]. Fumigating *Rubus idaeus* L. fruits with allyl isothiocyanate decreased the level of vitamin C. In *Fragaria x ananassas* Duch. fruits, garlic, and rosemary essential oil treatments did not significantly change the level of ascorbic acid compared to that in the non-treated samples [34]. As mentioned in Section 2.1., in that study essential oils were applied in a liquid form. Shortly after the treatment, the crops were washed; hence, contact with the essential oils and their aromatic compounds was very short. Thymol and eugenol vapors effectively enhanced the properties of modified atmosphere packages used for treatment of *Vitis vinifera* L. fruits, and they slowed down the loss of ascorbic acid significantly [29]. After 14 days of storage, table grapes fumigated with 150 µL of eugenol possessed levels of vitamin C content two times higher than in *Vitis vinifera* L. stored in MAP without any additional compounds. After the next 14 days, this difference increased—eugenol-treated fruits had about 55 mg of vitamin C/100 g while the control samples had about 20 mg/100 g.

From the group of carotenoids, the most common and beneficial for human health are lutein, lycopene, and β-carotene. The total carotenoid content in *Lactuca sativa* L. var. *longifolia* fumigated with methyl jasmonate before harvest remained at the same level, while non-treated samples suffered a decrease [14]. Additionally, methyl jasmonate vapor did not significantly affect the composition of β-carotene and lutein in lettuce, and it allowed it to maintain a constant level, whereas in the control sample, a loss was observed after 6 and 8 days of storage. The level of lycopene, which is a characteristic substance present in *Solanum lycopersicum* L. fruits, decreased dramatically after 1 week of storage due to the vapor of oregano essential oil [53], but the study did not show significant differences between the treated and control samples after 1 week of storage. However, in the following week, the concentration of lycopene increased from 150 to 350 mmol/g of fresh weight, obtaining a value significantly higher than that in the non-treated sample. This study did not show a statistically significant influence from oregano essential oil on lutein or β-carotene concentration in the samples.

Essential oils, however, influenced the carotenoid content in *Prunus persica* var. nucipersica fruits [19]. While the non-treated samples suffered a drop in the amount of carotenoids by about 14% after 14 days of storage, the drop was about 5% in samples treated with 10% thyme oil and 2% in those treated with 1% thyme oil. The trend was maintained through the next 2 weeks, with the concentration of carotenoids decreasing in all samples. Additionally, after 4 weeks, vapors of 10% savory oil significantly influenced the carotenoid content in the treated sample, which was higher than in the control sample but lower than in the thyme essential oil-treated sample. The differences were not that visible for *Prunus persica* L. fruits, as the results were not statistically significant. Loss of total carotenoid content as well as of β-carotene, lutein, and lycopene is due to oxidation processes that occur with light and heat exposure [56].

## 4. Sensory Attributes

Essential oils’ chemical composition determines their specific aromas and properties. Every constituent influences the final characteristic to a greater or lesser extent. EOs have been used in food technology for years as food additives, mainly as aromas and flavors but sometimes also as functional ingredients [57]. High volatility and the ability to penetrate tissues may cause changes in crops’ scent and taste after fumigation. Therefore, the final sensory properties of the crops might be unacceptable to customers. The below-mentioned studies present different aspects of sensory attributes that were subjectively evaluated by panelists (assessors) according to the researchers’ scales.

The off-odor of *Lentinus edodes* L. fruiting bodies fumigated by clove, cinnamaldehyde, and thyme oils was assessed as slightly less intense than that in control samples during 5, 10, 15, and 20 days of storage [17]. The same trend was observed for other evaluated properties; gill discolorations were less intense in fumigated samples than in the controls, and the number of dark zones was much lower—especially in samples treated with cinnamaldehyde and thyme essential oil. Similarly, the uniformity of gills and caps was more substantial in fumigated mushrooms. All of the results suggest that cinnamaldehyde was able to preserve the organoleptic attributes of *Lentinus edodes* L. fruiting bodies for a longer time and, therefore, retard the sensory decay.

In the case of *Avena sativa* L. (oat) seeds treated with cinnamon, thyme, clove, or lemongrass (*Cymbopogon Flexuosus* L.) essential oil vapors, the intensity of odors was much higher than in the non-treated samples, and in all of them, except for the lemongrass essential oil-treated sample, the treatment caused a decrease in odor acceptability [58]. For the sample fumigated with the lemongrass essential oil, the overall odor intensity was equal to 81.1, which was higher than the level for the control sample. What is more, the scent of lemongrass was easily distinguished by the assessor. Therefore, it can be concluded that even though the customers did recognize a strong odor of essential oil used in the treatment, it was favorable and increased the acceptability of the product. On the other hand, the clove scent, which was also very intense, was effortlessly identified in the clove essential oil-treated samples and diminished the seeds’ acceptability. However, it was not that simple to distinguish specific scents of thyme and oregano. They were confused with each other, and the acceptability of those samples was poor. The taste evaluation of those samples showed that essential oils were highly perceptible, and they were scored poorly—the lowest score was given to the oregano oil-treated sample, for which the acceptability was 25.1 while for the control sample, it was 63.7. In both oregano and thyme essential oil-treated samples, bitter and pungent tastes were perceptible. The best results from the treated samples were provided by the lemongrass essential oil-treated sample, with an acceptability level of 59.3, and it was due probably to the sweetness noticeable in a level similar to that of the control sample. Overall, all of the samples were assessed lower than the controls after 14 days of storage, and after 14 more days, samples fumigated with both concentrations of thyme oil yielded slightly higher results.

A high concentration of thyme essential oil, together with low and high concentrations of savory essential oil, caused an increase in off-aromas perceptible in *Prunus avium* L., (cv. “Ferrovia”) fruit samples treated with vapors of these oils two times after 14 days of storage [54]. Interestingly, the following 14 days of storage resulted in a decrease in off-odors in the samples fumigated with both concentrations of thyme oil—from 13 and 40 to 7 in both cases, while for savory essential oil-treated samples, it increased from 40 to 60 and 67 for the low and high concentrations, respectively. When it comes to taste evaluation, the sample treated with a low concentration of thyme essential oil possessed the highest number of off-flavors (40%) after 14 days of storage, and this score dropped after the next 14 days (to 13%) while the control sample was evaluated at 13% and 6% for 14 and 28 storage days, respectively. For the rest of the samples, the score was equal to 27% and, then, at 20% for a high concentration of thyme essential oil and 33% for both concentrations of savory essential oil. What is more, all the samples had similar scores for sweetness, acidity, and bitterness.

The assessment of sensory parameters, which included taste, texture, off-flavors, and overall acceptability of two cultivars of *Persea americana* Mill. fruits fumigated with thyme essential oil, showed that treatment resulted in much higher scores than for non-treated control fruits [59]. On the other hand, mugwort (*Artemisia nilagirica* L.) essential oil did not seem to influence *Vitis vinifera* L. grapes’ scent when a high concentration of essential oil was used [60]. Hence, the given score was 3.6 for low oil concentration treatments and 4.7 and 4.8 (out of 5) for higher treatment concentrations after storage. At the same time, a non-fumigated sample was scored at 1.5. The flavor of all the treated samples was assessed similarly: “good” when 300 µL of essential oil was used and between “fair/limited acceptability” and “good” when other concentrations were used. At the same time, a non-fumigated sample was scored at 2.2, which indicated flavor between “poor” and “fair/limited acceptability”. For the fruits’ firmness, the highest score, of 4.2, was given to those treated with 300 µL of essential oil, and on a hedonic scale, meant more than “good”. Similar but slightly lower results were assigned for lower concentrations of essential oil. At the same time, a non-fumigated sample was scored at 1.5, which meant between “very poor” and “poor”. It was probably connected with the fact that fumigated samples also had higher scores for “sweetness” and “juiciness” compared to the control sample. As a result, the essential oil vapor-treated samples were scored between 4.3 and 4.7 for overall acceptability, which was close to that of freshly picked fruits. Therefore one can conclude that mugwort oil had a positive influence on the sensory parameters of *Vitis vinifera* L. fruits. A similar conclusion could be made for the goldenrod essential oil vapor treatment of *Fragaria x ananassas* Duch. fruits, which helped to preserve the overall quality of fruits at a high level compared to that of the non-treated samples [41].

For *Fragaria x ananassas* Duch. treated with the vapor of thymol, the overall score of sensory reception was higher for higher concentrations of the substance [30]. As sensory properties, the researchers referred to aroma, taste, firmness, appearance, and texture of the fruits. When concentrations of 500 µL and 1000 µL were used, the score did not drop below 5 through the entire storage period, whereas the control sample was rated at 1 on the 6^th^ day. This score was maintained during the rest of the experiment.

Lack of influence on crop odor or aroma was observed in *Fragaria x ananassas* Duch. fruits treated with rosemary essential oil—the odor was scored on a level similar to that for the control samples [34]. Otherwise, when garlic essential oil was used in the same research, a strong garlic odor was perceptible in *Fragaria x ananassas* Duch. fruits, so it received 3 out of 5 points. However, when it comes to the taste evaluation, the highest level was reached by samples fumigated with garlic essential oil, and it was equal to 3.5 out of 5, while the sample fumigated with rosemary essential oil and the control sample were together scored at 2.5. All of the samples were evaluated similarly for flavor, and the overall evaluation scores were also almost equal for all fruits. As mentioned in Section 2.1., in this study essential oils were applied in liquid form. Shortly after the treatment, the crops were washed, and hence, the contact with essential oils and their aromatic compounds was very short.

It was also found that treating *Myrica rubra* Seib and Zucc. fruits with vapors of methyl jasmonate can help to maintain flavor, firmness, visual appearance, color, juiciness, and taste [25]. A comparison of the use of methyl jasmonate as an additive to its use in a modified atmosphere package and fumigation showed that both treatments caused an increase in the positive visual appearance of *Carica papaya* L. fruits [61]. Although the quality dropped during storage, it remained higher than the levels observed in the control sample. Using methyl jasmonate together with MAP helped to maintain high quality for a longer time, and the score after 32 days of storage was the highest among all the samples. Combining MAPs with essential oil constituents thymol and eugenol also resulted in higher levels of fruit and rachis aspects, firmness, and crunchiness in *Vitis vinifera* L. fruits [62]. In all of those categories, treated fruits were rated higher than the non-treated samples. However, treated samples received poor results for juiciness and sweetness in the context of taste aspects. There were no significant differences between the samples in sourness assessment.

Some studies suggested that changes in sensory properties might be caused by modification of metabolic patterns due to essential oil exposure [63]. The conclusion made by most of the researchers was that treatment with essential oils might cause an intensive change in the odor and taste of crops. It could be unfavorable; hence, the positive effects in prolonging the shelf-life of crops should be balanced with the sensory parameters. Unfortunately, not all researchers conducted sensory evaluations. The success of using essential oils as natural preservatives depends on consumer acceptance. Lack of acceptance disqualifies crops as food products, no matter the positive influence on quality properties or resistance to pathogens.

## 5. Physicochemical Parameters

This section describes the effects of essential oils fumigation of crops’ physicochemical properties: fruits firmness, the parameters of crops’ color and the weight loss during the storage. 

### 5.1. Firmness

Firmness is one of the indicators of crop freshness and quality. It is connected with the resistance to pathogens responsible for producing enzymes that cause fruit softening and decreases in firmness [64]. It was proven that fumigation with garlic and rosemary essential oils helped to maintain higher firmness in *Fragaria x ananassa* Duch. fruits than in non-treated samples [34]. Similar tendencies were visible in *Actinidia deliciosa* L. (kiwifruit) fruits treated with cinnamaldehyde and citral [65], *Lentinus edodes* L. and *Agaricus bisporus* L. fruiting bodies treated with clove essential oil, cinnamaldehyde, and thyme essential oil [17,18] or peppermint essential oil [15], *Solanum lycopersicum* L. treated with oregano essential oil [53], and *Morus alba* L. fruits treated with allyl isothiocyanate [43]. In *Prunus armeniaca* L. (cv. “Harglow”) (apricot) fruits inoculated with conidia of *Monilinia fructicola* and fumigated with thymol, firmness was lower than in non-treated samples, but only when the concentration of thymol was higher than 3 mg/L [66]. This study showed that the higher concentration of thymol, the higher the value for firmness. A slightly different situation was observed for *Citrus limon* L. fruits treated with peppermint and lavender (*Lavandula angustifolia* L.) essential oils—higher firmness was obtained using a higher concentration of mint essential oil and a lower concentration of lavender essential oil [67]. Nonetheless, in this study, the researchers stored the containers with *Citrus limon* L. fruits in a dark place with no access to sunlight or artificial light. That does not fully recreate the conditions in stores or markets where the light is intense and crop produce is exposed to it for many hours. Studies show that exposure to light might decrease the storage life of fruits by, among others, speeding the loss in firmness [68]. Therefore, the results of that study did not meet the conditions for actual food storage.

In all cases, the firmness of treated samples was higher, not only compared to the untreated control sample but also higher than their initial values. The influence of essential oils and their components used together with modified atmosphere packages was also examined in several studies. M. Serrano et al. [69] explored how the addition of eugenol, thymol, eucalyptol, and menthol affected *Prunus avium* L., (cv. “Ferrovia”) fruits stored in MAPs. This study showed that eugenol, thymol, and menthol assured the maintenance of high levels of fruit firmness, and the best results were achieved with the eugenol-treated samples. Although the menthol-treated samples and the controls (fruits stored in MAP without additional substances) possessed similar firmness values at the beginning of the storage period, after 9 days, when the firmness of the control fruits had dropped significantly, the menthol-treated samples still showed the same level of this parameter.

Poor results were visible for eucalyptol-treated samples, in which firmness was significantly lower than for the control sample and rapidly dropped after 13 days of storage. In turn, thymol and eugenol enhanced the firmness of *Vitis vinifera* L. fruits stored in the modified atmosphere package; however, increasing the thymol concentration caused a small drop in firmness, whereas increasing the eugenol concentration resulted in a higher firmness value [29]. Using both of those substances together with carvacrol as a mixture to fumigate *Vitis vinifera* L. fruits covered in clear films assured a much higher level of fruit firmness in comparison with the non-treated samples but also slightly higher than fruits covered in films without additional treatment [70]. Promising results from thymol treatment were also confirmed in a study that used thyme essential oil to enhance MAP in which two cultivars of *Persea americana* Mill. fruits were stored [31]. Although MAP alone assured a high value of firmness, the addition of thyme essential oil yielded even better results. The researchers suggest that this might be connected with the improved control of anthracnose in fruits stored in MAP with the addition of essential oil.

### 5.2. Color Parameters

Changes in the color parameters of food products occur due to maturation, decay, ripening, enzyme activity, and changes in antioxidant compound concentration [71,72]. According to Commission Internationale de l’Eclairage (CIE), which defined the L * a * b * color space, the L * (lightness) parameter corresponds to the ratio between reflected and absorbed light [73] and is an indicator of discoloration. Chromaticity coordinates a * and b * indicate red-green and yellow-blue coordinates, respectively [74]. However, L * C * h color space indicates L * as lightness, C * as chroma, and h as hue angle. Therefore, chroma corresponds to the brighter or duller color, and hue angle corresponds to the attribute of hue.

Considering mushrooms, the lightness parameter is an indication of acceptability and edibility, and it is an essential parameter for consumer acceptance. According to Gormley [75], the lowest value of the lightness parameter for mushrooms accepted by wholesalers is 80, and by consumers, is 69. Studies on *Agaricus bisporus* L. fruiting bodies treated with essential oil fumigation show that this treatment may delay decreases in lightness. The darkening of *Agaricus bisporus* L. fruiting bodies was significantly delayed by clove essential oil, cinnamaldehyde, and thyme essential oil, and what is more, L * of samples fumigated with cinnamaldehyde did not drop below 80 through the entire storage period [18]. All of the treated samples possessed a higher value for lightness than non-treated ones. Similarly, fumigation using 4-methoxy cinnamic acid [16], methyl jasmonate [26], and peppermint essential oil [15] enabled the high values of L * to be maintained for a longer time. Therefore, volatiles may be considered as color protectors of *Agaricus bisporus* L. fruiting bodies.

For blue-black *Vitis vinifera* L. fruits, the initial color is connected with the anthocyanin content [76]. Additionally, some cultivars of *Vitis vinifera* L. fruits (white-green) are characterized by green color due to the presence of chlorophyll [77]. Some papers indicate that storing those in modified atmosphere packages with essential oil vapors helps to maintain their initial color parameters. Guillén et al. [70] showed that using a mixture of eugenol, thymol, and carvacrol slows down the decrease in lightness and chroma, probably due to the delay in chlorophyllase activity, which is responsible for chlorophyll degradation. Additionally, a smaller increase in the hue angle value and a smaller decrease in lightness were obtained when thymol and menthol were added to MAPs in which the *Vitis vinifera* L. fruits were stored [62]. The addition of eugenol and especially thymol reduced changes in L* and a* parameters compared to the control samples but did not influence the increase in b* value [29]. Thyme and savory essential oil treatments of *Prunus avium* L. (cv. “Ferrovia”) fruits, showed no significant differences between treatments when fruits were cold-stored. However, for storage at 20°C, the highest lightness values were observed in samples fumigated with low and high savory essential oil concentrations and with a low concentration of thyme essential oil [54]. Additionally, the chroma value was reduced in *Prunus avium* L. (cv. “Ferrovia”) fruits stored in MAPs with the addition of eugenol, thymol, and menthol [69]. However, when eucalyptol was added, the decrease in the chroma value was faster and more robust than observed in all other samples, treated and non-treated. Changes were also more rapid when samples were stored for one day at 20 °C.

Similar results were obtained in *Morus alba* L. fruits treated with allyl isothiocyanate, which slowed the rate of decrease in a *, b *, and L * values in comparison to control samples [43] and in *Solanum melongena* L. (eggplant) fruits treated with eugenol, which slowed the rate of decrease in L * [78]. In *Actinidia deliciosa* (A. Chev) (kiwifruit) fruits, the values for lightness and chroma increased in all samples, treated and non-treated, but fumigation with cinnamaldehyde and citral resulted in boosting the rate of this increase. For *Prunus persica* L. and *Prunus persica* L. var. nucipersica fruits, the increase in hue angle was connected with ripening because the fruits were becoming more yellow. A slight increase in hue angle was observed in *Prunus persica* L. var. nucipersica fruits treated with savory essential oil and stored in a normal atmosphere; however, no differences were observed in *Prunus persica* L. fruits treated with savory and thyme essential oil [19]. In *Rubus idaeus* L. fruits treated with tea tree essential oil and methyl jasmonate, the value of a * was higher than in a control sample [10]. However, the highest value of L * was assured by allyl isothiocyanate followed by methyl jasmonate, while for a tea tree essential oil-treated sample, lightness was lower than in a control sample. Those results were based on determination of the b * value because a high b * corresponds to darker samples and a low b* corresponds to lighter samples. So, the highest value for b * was obtained with allyl isothiocyanate and the lowest with tea tree essential oil. Using methyl jasmonate as an additive to a modified atmosphere package in which *Carica papaya* L. (cv. “Sunrise”) fruits were stored resulted in suppressing the increase in b* value in comparison to using methyl jasmonate alone, resulting in more yellowish fruits [61]. A study on *Persea americana* Mill. fruits stored in MAP with and without the addition of thyme essential oil showed that the presence of essential oil resulted in higher lightness values than those for other samples; however, the value of hue angle remained on the same level in all samples [31].

Because some essential oils and their components sometimes influence the content of anthocyanins as described in this paper, they may also strongly affect the color parameters of some fruits. For example, *Fragaria x ananassas* Duch. fruits treated with methyl jasmonate experienced a substantial increase in a* and chroma (which indicates the intensity of a color) [79]. As a result, the values for lightness and hue angle decreased. An increase in chroma and decreased lightness caused by methyl jasmonate treatment was also observed in *Malus domestica* L. (apple) fruits treated with this volatile [80], as they are also rich in anthocyanins [81].

### 5.3. Weight Loss

Weight loss is a parameter connected with the metabolic activity of fruits and it is specifically associated with the process of deterioration [82]. Some studies show that treatments with essential oils may reduce the process of dehydration and, as a result, maintain the freshness of food products. For example, thyme and savory essential oils reduced weight loss in *Prunus persica* L. and *Prunus persica* var. nucipersica fruits [19]. As suggested in this study, essential oils cause a coating to form on the surface of the fruit due to fumigation, thus modifying gas permeation. Although weight loss increases during storage, volatiles significantly reduced it in *Agaricus bisporus* L. fruiting bodies that were treated with cinnamaldehyde, thyme, or clove essential oils; of those three, the most effective was clove essential oil, followed by cinnamaldehyde and thyme essential oil [18]. A similar effect was reported in Hu et al. [16], a study that used the volatile 4-methoxy cinnamic acid, and in Qu et al. [15], where peppermint essential oil was used. The percentage of open caps is another factor that characterizes mushroom dehydration and serves as a measure of the mushrooms’ freshness index. This indicator is assessed by the development of the umbrella-like shape of the caps followed by the detachment of the veils [18]. As the water in a mushroom is lost, the cohesive forces of hydrophilic molecules that keep the cap in position decrease [52]. Therefore, if the volatile can positively influence weight loss, it can also be responsible for a drop in the percentage of open caps. Such a mechanism was observed in both mentioned studies. In Gao et al. [18], the lowest value for this factor was obtained with the cinnamaldehyde-treated sample, followed by thyme and clove essential oils. All of them maintained lower values compared to the control, non-treated sample. Moreover, 4-methoxy cinnamic acid caused a significant drop in the percentage of open caps in *Agaricus bisporus* L. fruiting bodies compared with control, non-treated samples [16].

Reductions in weight loss due to fumigation were observed also in *Prunus avium* L. (cv. “Ferrovia”) fruits treated with thyme and savory essential oils (with small differences between treatments ) [54], *Fragaria x ananassas* Duch. fruits treated with thymol [30], and *Solanum melongena* L. fruits treated with eugenol [78]. However, garlic and rosemary essential oil treatments did not yield any significant differences between the treated and nontreated *Fragaria x ananassas* Duch. fruits [34], and the same was observed for *Carica papaya* L. fruits treated with lemongrass essential oil [83] and *Fragaria x ananassas* Duch. fruits treated with goldenrod essential oil [41]. In Sumalan et al. [67], three essential oils were used in two concentrations to fumigate *Citrus limon* L. fruits: mint, basil (*Ocimum basilicum* L.), and lavender essential oils. The results showed that a high concentration of mint essential oil and low concentrations of basil and lavender essential oils reduced the weight loss slightly, but a low concentration of mint essential oil and high concentration of basil essential oil caused a slight increase in weight loss. However, as the fruits were stored only in a dark place, one should remember that these conditions also influenced the weight loss, as shown in a study by Tombesi et al. [68]. The results from a high concentration of lavender essential oil showed no significant differences between the treated and nontreated samples. Additionally, treatment with oregano essential oil caused an increase in weight loss in *Solanum lycopersicum* L. fruit compared to the non-treated sample after 1 week of storage [53]. On the other hand, citronella essential oil caused a decrease in weight loss in *Solanum tuberosum L.* (potato) tubers in both treatments—single-phase and dual-phase, with a more substantial effect from the single-phase treatment [84].

In terms of using volatiles in combination with modified atmosphere packages, Sellamuthu, Mafune, et al. [31] showed that MAP overshadowed the effects of fumigation because both samples stored in MAP and samples stored in MAP with thyme essential oil suffered almost identical weight loss, albeit much less than non-treated samples. Similar results were shown for *Vitis vinifera* L. fruits stored in the modified atmosphere with a combination of eugenol and thymol; although all of the treated samples lost much less weight, there were no significant differences between treatments with or without volatiles [70]. The reduction in weight loss in *Carica papaya* L. fruits was also attributed to MAP instead of methyl jasmonate [61]. Nevertheless, thymol and eugenol showed some positive effects in reducing weight loss in *Vitis vinifera* L. fruits [29]. Additionally, in Valverde et al. [62], it was shown that the addition of menthol, thymol, and eugenol significantly enhanced the reduction in dehydration of *Vitis vinifera* L. fruits assured by modified atmosphere packages. The same set of volatiles significantly reduced the weight loss in *Prunus avium* L. fruits, but it was not reduced when eucalyptol was used [69].

It is possible that essential oil vapors, which are hydrophobic, act similarly to coatings and can extend the shelf-life of crops due to the physical barriers they produce [85,86]. Hence, they can reduce the water loss and delay the side process of respiration. It is also assumed that treatments with essential oils and their components influence transpiration and respiratory metabolism [54].

## 6. Effects on Organic Acids, and Macronutrient Compounds

The amounts of sugars, organic acids, and proteins contribute to the overall acceptance of products due to their organoleptic qualities—mainly flavor. The levels of these compounds are dependent on the enzymes and metabolites that take part in the metabolism of carbons during the ripening of fruits [87].

In *Rubus idaeus* L. fruits, the main organic acid is citric acid; after harvesting, its concentration in the fruits drops significantly. Although the malic acid concentration in *Rubus idaeus* L. fruits is lower, the decrease due to harvest is more substantial [10]. The study on those fruits treated with natural volatile compounds showed that methyl jasmonate, allyl isothiocyanate, and tea tree essential oil might be efficient in maintaining higher levels of organic acids and slowing their decreases [10]. Additionally, regarding sugar content, fructose, sucrose, and glucose levels in fruits treated with methyl jasmonate and tea tree essential oil were higher than those in non-treated fruits stored in the same conditions. Using methyl jasmonate as a fumigant for the *Agaricus bisporus* fruiting body delayed the decrease in sugar content; however, the effect was more substantial for a higher concentration of this volatile [26].

Similarly, a high concentration of methyl jasmonate resulted in higher protein content than in the control sample and in the sample treated with a lower concentration of methyl jasmonate. The results for fumigation with methyl jasmonate showed that it can increase the levels of malic, citric, and quinic acids in comparison to the non-treated samples [61].

In *Fragaria x ananassas* Duch. fruits fumigated with menthol, thymol, and eugenol, the contents of sugars and organic acids were higher than in the non-treated samples [11]. It turned out that menthol was the most effective in sustaining a high level of fructose; thymol assured the highest level of glucose, and eugenol assured the highest levels of sucrose, malic acid, and citric acid.

A study on *Vaccinium corymbosum* L. treated with essential oils showed that the most effective substances for maintaining high organic acid and sugar levels were carvacrol, anethole, perillaldehyde, and cinnamaldehyde [13]. Compared to control samples in that study, cinnamic acid-treated samples possessed higher levels of glucose but lower levels of fructose and citric acid, and linalool-treated samples possessed higher levels of fructose and glucose but lower levels of citric acid. The *p*-cymene treatment was not effective in any of those evaluations. Another study on *Vaccinium corymbosum* L. fruits showed that during storage, the content of fructose and glucose in samples treated with allyl isothiocyanate rose until the 10th day of storage, and then, both levels started to drop [47]. After 14 days of storage, the glucose level in those samples was still higher than the initial level, but the level of fructose was lower. The decrease in citric acid during storage, on the other hand, was accelerated due to allyl isothiocyanate treatment. Hence, the citric acid content was lower in the treated samples than in the non-treated through the whole storage period.

The experiment on *Solanum lycopersicum* L. fruits fumigated with oregano essential oil showed that the treatment might induce a significant increase in glucose, fructose, and total sugar content after 2 weeks of storage, but has no significant influence on organic acid content [53].

In contrast to those results, two studies on *Vitis vinifera* L. fruits treated with essential oil vapors indicated a decrease in sugar content in fruits due to treatment. In *Vitis vinifera* L. fruits fumigated with mugwort essential oils, total sugar content in treated berries was lower than in non-treated control samples [60]. What is more, the difference was larger for a higher concentration of essential oil. A similar situation was observed when MAPs with the addition of thymol and eugenol were used; the highest contents of glucose and fructose were recorded in the control samples in both types of storage—cold and normal (shelf-life storage) [29]. In all samples, levels of fructose and glucose rose during storage. Regarding organic acids in this experiment, tartaric acid was determined as it was the principal constituent in *Vitis vinifera* L. fruits. This compound’s concentration also dropped during storage; however, the decrease was attenuated by the addition of thymol and eugenol, and this tendency was observed for both cold storage and shelf-life storage. Higher concentrations of volatile components assured better results.

## 7. Decay Parameters

During storage, plants may suffer from microbial attacks or diseases, and their resistance to those determines their shelf-life. Therefore, this section discusses decay-distinguishing factors and the influence of essential oil fumigation on inhibition of food spoilage.

As mold is one of the most common types of microbial attacks on fruits, the decay incidence may be defined by observing mold on the surface of the fruit. It was shown for *Myrica rubra* Seib and Zucc. fruits that after 12 days of storage, about 33% of fruits had decayed, while depending on the volatile used as a fumigant, this number was lowered by 7% (for linalool) to 14% (for carvacrol) [33]. Another study on those fruits tested methyl jasmonate as a fumigant, and it was shown that the effects on decay are significantly dependent on the concentration of this volatile, because after 12 days of storage, high concentrations (1000 and 100 µmol/L) were not effective in comparison to the control sample, whereas a concentration of 10 µmol/L resulted in about 27% less decayed berries [45]. Additionally, 10 µmol/L of methyl jasmonate was used to fumigate *Solanum lycopersicum* L. fruits, which resulted in 23.5% of overripened fruits after storage for 4 weeks at 5°C, while all of the control fruits were spoiled by that time [7]. Although it seems that lower concentrations of methyl jasmonate are more effective than higher concentrations, that is not the case for other volatiles. For example, a study on thymol-treated *Fragaria x ananassas* Duch. fruits showed that the higher the concentration of thymol, the stronger the effect of decay inhibition [30]. Among the volatile compounds thymol, menthol, or eugenol, thymol assured the best results in reducing the number of decayed fruits, followed by menthol and eugenol [11]. Treatments with any of these substances resulted in a much lower final amount of overripe fruit than when no processing was performed. After 14 days of storage, approx. 80% of control fruits, 45% of eugenol-treated fruits, 40% of menthol-treated fruits, and 30% of thymol-treated fruits were spoiled.

Another substance effective in retarding decay was allyl isothiocyanate, used in *Morus alba* L. fruit treatment; it completely inhibited mold growth throughout the entire storage period [43]. Allyl isothiocyanate was also shown to be more effective than methyl jasmonate and tea tree essential oil in reducing the severity of decay in *Fragaria x ananassas* Duch. and *Rubus* L. fruits [12]. Although all of the substances used as fumigants decreased the percentage of fruits showing fungal symptoms, the effects of allyl isothiocyanate were the strongest. Additionally, a mixture of eugenol, thymol, and carvacrol reduced the percentage of decayed *Vitis vinifera* L. fruits in baskets thermo-sealed with 2 films to about 5% [70].

The effectiveness of goldenrod essential oil vapor was confirmed by *Fragaria x ananassas* Duch. fruit treatment, where the development of decay was reduced by 35% after 3 days of storage and by 25% after 4 days of storage compared to the non-treated control fruit [41]. The index of decay was also evaluated for samples artificially inoculated by 15 µL of *Botrytis cinerea* suspension. As a result, the decay index decreased by 76% compared to the control samples (inoculated with spores but not fumigated). Therefore, goldenrod essential oil seems to be effective in grey mold inhibition. The severity of decay also decreased due to oregano essential oil treatment of *Solanum lycopersicum* L. fruit (by 59%, compared with the control) [53], methyl jasmonate treatment of *Carica papaya* L. fruit [61], and decay incidence was decreased by a combination of modified atmosphere packages with thymol, menthol, and eugenol in *Vitis vinifera* L. fruit [29,62]. The rot incidence in non-treated *Prunus persica* var. nucipersica fruit reached 23.3%, while it was 7.3% on fruit treated with 1% thyme essential oil, 9.3% on fruit treated with 10% thyme essential oil, 14.6% on fruit treated with 1% savory essential oil, and 4% on fruit treated with 10% savory essential oil [19]. The researchers also analyzed *Prunus persica* L. fruits, and the results were not that promising. Whereas 99.3% of the control fruits were decayed, 91.4% of those treated with 10% savory essential oil were decayed, and the rot incidence in *Prunus persica* L. fruits treated with 1% of savory essential oil was only 8% smaller. Applying thyme essential oil gave slightly better results: decay was observed in 71.9% and 48.7% of the fruits fumigated with 1% and 10% thyme essential oil, respectively.

Božik et al. [58] showed how various essential oils in different concentrations affect the percentage of infected seeds and sporulated *Avena sativa* L. seed samples. The results suggested that in terms of decreasing the number of infected seeds, the most effective treatments were high lemongrass and oregano essential oil concentrations (500 µL/L) and moderate (250 µL/L) lemongrass essential oil concentrations, whereas lower concentrations yielded decreased effectiveness. Low concentrations (125 µL/L) of clove and ginger essential oils were non-effective and gave results similar to non-treated control samples.

Additionally, Ziedan and Farrag [88] assessed the usefulness of peppermint and basil essential oils in inhibiting typical fungal growth in *Prunus persica* L. fruits. The results showed that *Prunus persica* L. fruits inoculated with *Rhizopus stolonfier* and *Monilinia fructicola* did not suffer decay when fumigated with 3 or 4 mL of peppermint essential oil and 4 mL of basil essential oil. Using 3 mL of basil essential oil completely inhibited the decay of *Monilinia fruticola* inoculated *Prunus persica* L. fruits, but in the case of *Rhizopus stolonfier* inoculated samples, 2% suffered decay. Similarly, cinnamon essential oil vapor decreased the fungal decay index for *Vitis vinifera* L. inoculated with *Botrytis cinerea* in all used concentrations [48]. In *Citrus limon* L. fruits inoculated with *Penicilium digitatum*, the mycelium growth was measured as a diameter of fungal decay [67]. The results showed that the largest diameter occurred in non-treated samples, then in fruit samples treated with 43.0 µL/L of lavender essential oil, 91.5 µL/L of mint essential oil, 23.0 µL/L of basil essential oil, 183 µL/L of mint essential oil, 46 µL/L of basil essential oil, and 86 µL/L of lavender essential oil, respectively. The concentrations were selected according to the previously determined minimum inhibitory dose. Methyl jasmonate significantly lowered the incidence of decay and decreased the lesion diameter caused by inoculation of *Prunus persica* L. fruits with *Penicillium expansum, Botrytis cinerea, and Rhizopus stolonifera* [24]. The study noted that due to methyl jasmonate fumigation, the decay incidence on *Prunus persica* L. fruits after 2 days of storage was 77.8% lower than in control samples, and after 4 days of storage, 25% lower. However, after 6 days, decay incidence was equal for both treated and non-treated samples. On the other hand, the lesion diameter at that time was still lower for fumigated samples.

Fruit decay might occur due to different fungal infections leading to various types of anthracnose and stem-end rot. The severity and incidence of anthracnose and stem-end rot were tested in two different *Mangifera indica* L. cultivars treated with clove, thyme, and cinnamon essential oils [32]. All of the tested essential oils significantly reduced disease incidence and severity; however, thyme essential oil controlled it more strongly than the clove and cinnamon essential oils.

Thyme essential oil assured the most promising results in inhibiting anthracnose decay in two cultivars of *Persea americana* Mill. fruits compared to citronella or peppermint essential oils [28]. Still, all of the volatiles reduced anthracnose incidence and severity. What is more, thyme essential oil required a lower concentration than the other essential oils—66.7 µL/L of thyme essential oil was more effective than 106 µL/L of citronella or peppermint essential oils. The effectiveness of thyme essential oil was also confirmed in *Persea americana* Mill. fruits in a study by Bill et al. [59], which tested fumigation of artificially inoculated *Persea americana* Mill. cultivars to assess its influence on the incidence and severity of anthracnose. The results showed that 96 µL/L successfully reduced the disease incidence by 72% in cv. “Hass” and by 64.2% in cv. “Ryan” and reduced the disease severity by 16.9% in cv. “Hass” and 9.9% in cv. “Ryan”, in comparison to the non-treated control fruits.

Moreover, the addition of thyme essential oil to the modified atmosphere package significantly reduced the incidence and severity of anthracnose in “Fuerte” and “Hass” cultivars of *Persea americana* Mill. even in comparison with the use of MAP alone [31]. As the atmosphere composition in MAP was identical for both samples (with and without thyme essential oil), it can be concluded that it was the thyme essential oil that controlled the anthracnose development. Another study that tested the feasibility of fumigation with two essential oils (garlic and rosemary essential oils) against *Colletotrichum nymphaeae* inciting *Fragaria x ananassas* Duch. fruit anthracnose under greenhouse conditions showed strong reductions in both the severity and incidence of anthracnose by both essential oils, in comparison to the control [34]. Although garlic essential oil assured more robust results than rosemary essential oil, it did not show any significant differences compared to non-treated, non-inoculated control. Furthermore, basil, orange (*Citrus aurantium* L.), lemon, and mustard (*Sinapis alba* L.) essential oils used as fumigants to treat *Mangifera indica* L. fruits assured low levels of anthracnose incidence, which were lower with higher essential oil concentrations [3]. The most effective was basil essential oil used in a concentration of 100 ppm, which reduced anthracnose incidence to 5.6%, while for the control sample, the incidence was 100%. All of the considered studies confirmed the effectiveness of essential oils in fumigation processes as inhibitors of disease and rot development. It is a known fact that most of the phyto-phenolic compounds present in plants and their essential oils have antimicrobial properties [89]. This antifungal activity of essential oils is probably due to the presence of some essential oil components that can form hydrogen bonds with active enzymes, resulting in deactivation and, consequently, affecting the biosynthesis of toxins that cause plant diseases [90]. Those components are, among others, cinnamaldehyde, thymol, eugenol, carvacrol, trans-anethole, and camphor.

## 8. Conclusions

Many studies have broadly verified the bioactivity of essential oils. These oils have proven antifungal, antibacterial, and antioxidant properties, so it is natural to apply them as food preservatives. Retarding postharvest decay is one of the most significant challenges for modern society, which faces enormous food losses. There is also demand for the use of eco-friendly solutions, such as essential oils and their constituents, to prolong the shelf-life of food products. A significant number of studies have demonstrated the effectiveness of fumigation with essential oils in changing the biochemical, microbial, and physicochemical parameters of crops. Therefore, applying this technique and using a proper volatile in adjusted concentration for a specific fruit or vegetable may prolong the shelf-life and not only maintain but also improve the quality of food products (Table 1). However, some of the essential oils may negatively influence the organoleptic properties of fruits and vegetables. Unfortunately, no papers focusing on applying this technique on a technical scale were found. Additionally, there is still little information about the possible mechanism that stands behind those effects, and there are many different essential oils with effects in this context that were not studied. Moreover, the majority of studies are not recent, so most of the knowledge about fumigation with essential oils is older than 15 years. The biggest objection to the reviewed articles is that in most of them, the storage conditions did not recreate the conditions of shelf-life storage. Most of the researchers kept the samples in cold conditions or dark places, which do not reflect the conditions of shelf-life. Additionally, in many papers, the researchers indicated the initial concentration of essential oil in a container but did not take into account the fact that the concentration per sample would change when removing a sample from the container. Another significant fact is that proposing higher concentrations of rare and expensive essential oils would result in an enormous increase in food product prices.

Given that the reviewed studies had many objectives and only a small number are recent, this topic should be expanded in the future.

## Figures and Tables

**Table 1:** Presentation of volatiles that enhanced quality of analyzed fruits in studies.

Volatile	Parameter	Fruit	Reference
Methyl jasmonate	Total phenolics content ↑	*Prunus persica* L. *Myrica rubra* Seib and Zucc. *Agaricus bisporus* L. *Rubus idaeus* L.	[24] [25] [26] [9]
	PPO, POD activity ↑	*Prunus persica* L. *Myrica rubra* Seib and Zucc.	[24] [25]
	SOD activity ↑	*Rubus idaeus* L. *Prunus persica* L. *Agaricus bisporus*	[9] [24] [26]
	Ascorbic acid content ↑	*Rubus idaeus* L.	[9]
	Decay incidence * ↓	*Myrica rubra* Seib and Zucc. *Solanum lycopersicum* L. *Fragaria x ananassas* Duch. *Rubus* L. *Prunus persica* L.	[25,45] [7] [12] [12] [24]
	Sensory attributes ↑	*Myrica rubra* Seib and Zucc. *Carica papaya* L.	[25] [61]
	Decay severity ** ↓	*Rubus idaeus* L. *Carica papaya* L. *Prunus persica* L.	[10] [61] [24]
	Fructose, glucose, malic, and citric acids content ↑ Total sugars ↑	*Rubus idaeus* L. *Agaricus bisporus*	[10] [26]
	Total carotenoids, lutein, β-carotene content ↑	*Lactuca sativa* L. var. *longifolia*	[14]
Allyl isothiocyanate	Firmness ↑	*Morus alba* L.	[43]
	Malic acid content ↑ Decay severity ** ↓	*Rubus idaeus* L.	[10]
	Decay incidence * ↓	*Morus alba* L. *Fragaria x ananassas* Duch. *Rubus* L.	[43] [12] [12]
	Glucose and fructose content ↑	*Vaccinium corymbosum* L.	[27]
4-methoxy cinnamic acid	Ascorbic acid content ↑ Weight loss, percentage open caps ↓	*Agaricus bisporus* L.	[16]
Carvacrol	Total phenolics content ↑	*Myrica rubra* Seib and Zucc. *Vaccinium corymbosum* L.	[33] [13]
	Decay incidence * ↓	*Myrica rubra* Seib and Zucc. *Vitis vinifera* L.	[33] [69]
	Citric acid content ↑ Fructose and glucose content ↑	*Vaccinium corymbosum* L.	[13]
	POD, CAT, APX, SOD activity ↑	*Myrica rubra* Seib and Zucc.	[33]
Anethole	Total phenolics content ↑ Citric acid content ↑ Fructose and glucose content ↑	*Vaccinium corymbosum* L.	[13]
Menthol	Total phenolics content ↑ Fructose, glucose, and sucrose content ↑ Malic and citric acids content ↑	*Fragaria x ananassas* Duch.	[11]
	Decay incidence * ↓	*Vitis vinifera* L. *Fragaria x ananassas* Duch.	[29] [11,62]
	Firmness ↑	*Prunus avium* L. cv Ferrovia	[20]
Thymol	Total phenolics content ↑	*Fragaria x ananassas* Duch. *Vitis vinifera* L.	[11] [29]
	Ascorbic acid content ↑	*Fragaria x ananassas* Duch. *Vitis vinifera* L.	[30] [29]
	Malic and citric acids content ↑ Fructose, glucose, and sucrose content ↑	*Fragaria x ananassas* Duch.	[11]
	Decay incidence * ↓	*Fragaria x ananassas* Duch. *Vitis vinifera* L.	[11,30] [29,69]
	Tartaric acid content ↑	*Vitis vinifera* L.	[29]
	Sensory attributes ↑	*Fragaria x ananassas* Duch. *Vitis vinifera* L	[30] [62]
	Firmness ↑	*Prunus armeniaca* L. *Prunus avium* L. cv Ferrovia *Vitis vinifera* L. *Persea americana* Mill.	[66] [20] [29] [31]
	Weight loss ↓	*Fragaria x ananassas* Duch. *Vitis vinifera* L. *Prunus avium* L. cv Ferrovia	[30] [29] [20]
Citral	Firmness ↑	*Fragaria x ananassas* Duch.	[65]
Cinnamic acid	Glucose content ↑	*Vaccinium corymbosum* L.	[13]
Eugenol	Total phenolics content ↑	*Fragaria x ananassas* Duch. *Vitis vinifera* L.	[11] [29]
	Firmness ↑	*Prunus avium* L. cv Ferrovia *Vitis vinifera* L.	[20] [29]
	Sensory attributes ↑	*Vitis vinifera* L	[62]
	Decay incidence * ↓	*Fragaria x ananassas* Duch. *Vitis vinifera* L.	[11] [29,69]
	Ascorbic acid content ↑ Tartaric acid content ↑	*Vitis vinifera* L.	[29]
	Malic and citric acid content ↑ Fructose, glucose, and sucrose content ↑	*Fragaria x ananassas* Duch.	[11]
	Weight loss ↓	*Solanum melongena* L. *Vitis vinifera* L. *Prunus avium* L. cv Ferrovia	[77] [29] [20]
	Fructose and glucose content ↑	*Vaccinium corymbosum* L.	[13]
	Decay incidence ↓ POD, CAT, APX activity ↑ Total phenolics content ↑	*Myrica rubra* Seib and Zucc.	[33]
Perillaldehyde	Total phenolics content ↑	*Myrica rubra* Seib and Zucc. *Vaccinium corymbosum* L.	[33] [13]
	POD, CAT, APX activity ↑	*Myrica rubra* Seib and Zucc.	[33]
	Citric acid content ↑ Fructose and glucose content ↑	*Vaccinium corymbosum* L.	[13]
Tea tree EO *Melaleuca alternifolia* L.	Total phenolics content ↑ Ascorbic acid content ↑	*Rubus idaeus* L.	[9]
	Malic and citric acid content ↑ Glucose, fructose, and sucrose content ↑	*Rubus idaeus* L.	[10]
	Decay incidence * ↓	*Rubus idaeus* L., *Rubus* L.	[12]
Thyme EO *Thymus vulgaris* L.	Total phenolics content ↑	*Persea americana* Mill. *Agaricus bisporus* L. *Lentinus edodes* L *Mangifera indica* L.	[28] [18] [17] [32]
	Decay incidence * ↓	*Prunus persica* L. var. nucipersica *Prunus persica* L.	[19]
	Sensory attributes ↓	*Prunus avium* L. (cv. “Ferrovia”) *Avena sativa* L.	[54] [58]
	Sensory attributes ↑	*Lentinus edodes* L. *Persea americana* Mill.	[17] [59]
	Anthracnose severity and incidence ↓	*Mangifera indica* L. *Persea americana* Mill.	[32] [31,59]
	Weight loss ↓	*Prunus persica* L. var. nucipersica *Prunus persica* L. *Prunus avium* L. (cv. “Ferrovia”)	[19] [19] [54]
	POD, SOD activity ↑	*Persea americana* Mill. *Mangifera indica* L.	[28] [32]
	PPO activity ↑	*Agaricus bisporus* L.	[18]
		*Persea americana* Mill.	[31] [28]
	Ascorbic acid content ↑	*Prunus persica* L. var. nucipersica *Agaricus bisporus* L.	[19] [15]
	Total carotenoids content ↑	*Prunus persica* L. var. nucipersica	[19]
	Firmness ↑	*Agaricus bisporus* L. *Lentinus edodes* L.	[18] [17]
Cinnamaldehyde	Total phenolics content ↑	*Myrica rubra* Seib and Zucc. *Vaccinium corymbosum* L. *Agaricus bisporus* L. *Lentinus edodes* L.	[33] [13] [18] [17]
	POD, CAT, APX activity ↑	*Myrica rubra* Seib and Zucc. *Lentinus edodes* L.	[33] [17]
	PPO activity ↑ Weight loss, percentage open caps ↓	*Agaricus bisporus* L.	[18]
	Ascorbic acid content ↑	*Agaricus bisporus* L.	[16]
	Sensory attributes ↑	*Lentinus edodes* L.	[17]
	Firmness ↑	*Fragaria x ananassas* Duch. *Agaricus bisporus* L. *Lentinus edodes* L.	[65] [18] [17]
	Citric acid content ↑ Fructose and glucose content ↑	*Vaccinium corymbosum* L.	[13]
Cinnamon EO *Cinnamomum* *zeylanicum* L.	Total phenolics content ↑ POD activity ↑ Anthracnose severity and incidence ↓	*Mangifera indica* L.	[32]
	Decay severity ** ↓	*Vitis vinifera* L.	[48]
	Sensory attributes ↓	*Avena sativa* L.	[58]
Lavender EO *Lavandula angustifolia* L.	Firmness ↑ Decay severity ** ↓ Weight loss ↓	*Citrus limon* L.	[67]
Clove EO *Syzygium aromatic* L.	Total phenolics content ↑	*Lentinus edodes* L. *Mangifera indica* L.	[17] [32]
	Weight loss, percentage open caps ↓	*Agaricus bisporus* L.	[18]
	Anthracnose severity and incidence ↓ POD activity ↑	*Mangifera indica* L.	[32]
	APX, GR activity ↑	*Lentinus edodes* L.	[17]
	PPO activity ↑	*Agaricus bisporus* *Mangifera indica* L.	[18] [32]
	Sensory attributes ↓ Sensory attributes ↑	*Avena sativa* L. *Lentinus edodes* L.	[58] [17]
	Ascorbic acid content ↑	*Agaricus bisporus* L.	[15]
	Firmness ↑	*Agaricus bisporus* L. *Lentinus edodes* L.	[18] [17]
Oregano EO *Origanum vulgare* L.	Ascorbic acid content ↑ Lycopene ↑ Firmness ↑ Total sugars, glucose, and fructose content ↑ Decay severity ** ↓	*Solanum lycopersicum* L.	[53]
	Decay incidence * ↓	*Avena sativa* L.	[58]
Peppermint EO *Mentha piperita* L.	Total phenolics content ↑ SOD activity ↑ PPO activity ↑	*Agaricus bisporus* *Persea americana* Mill.	[15] [28]
	Decay incidence * ↓	*Prunus persica* L.	[87]
	Anthracnose severity and incidence ↓	*Persea americana* Mill.	[28]
	Firmness ↑	*Agaricus bisporus* L.	[15]
	Weight loss ↓ Decay severity **	*Citrus limon* L.	[67]
Basil EO *Ocimum basilicum* L.	Weight loss ↓ Decay severity **	*Citrus limon* L.	[67]
	Decay incidence *	*Prunus persica* L.	[87]
	Anthracnose incidence ↓	*Mangifera indica* L.	[3]
Rosemary EO *Rosmarinus officinalis* L.	Firmness ↑ Anthracnose severity and incidence ↓ Sensory attributes ↑	*Fragaria x ananassas* Duch.	[34]
Garlic EO *Allium sativum* L.	Firmness ↑ Anthracnose severity and incidence ↓ Sensory attributes (taste) ↑ Sensory attributes (scent) ↓	*Fragaria x ananassas* Duch.	[34]
Savory EO *Satureja montana* L.	Ascorbic acid content ↑ Total carotenoids content ↑	*Prunus persica* L. var. nucipersica	[19]
	Weight loss ↓	*Prunus avium* L. (cv. “Ferrovia”) *Prunus persica* L. var. nucipersica *Prunus persica* L.	[54] [19] [19]
	Sensory attributes ↓	*Prunus avium* L. (cv. “Ferrovia”)	[54]
	Decay incidence * ↓	*Prunus persica* L. var. nucipersica *Prunus persica* L.	[19]
Lemongrass EO *Cymbopogon citratus* L.	Decay incidence * ↓ Sensory attributes ↓	*Avena sativa* L.	[58]
Citronella EO *Cymbopogen nardus* L.	Total phenolics content ↑ POD activity ↑ Anthracnose severity and incidence ↓	*Persea americana* Mill.	[28]
	Weight loss ↓	*Solanum tuberosum* L.	[83]
Goldenrod EO *Solidago canadensis* L.	Decay severity ** ↓ Sensory attributes ↑	*Fragaria x ananassas* Duch.	[41]

↑ indicates the increase in this parameter due to treatment with volatiles. ↓ indicates the decrease in this parameter due to treatment with volatiles. * Decay incidence is expressed as the percentage of fruits showing decay symptoms, regardless of the term used in mentioned reference. ** Decay severity is expressed as the intensity of the decay visible on the fruits, regardless of the term used in mentioned reference.

## Data Availability

Not applicable.
